# Moderating Effects of the Low-Income Housing Tax Credit on Associations Between Race and Elevated Blood Pressure in Chicago

**DOI:** 10.1007/s11524-025-00983-y

**Published:** 2025-06-18

**Authors:** Tiffany H. Xie, Monica E. Peek, Corey E. Tabit, Elizabeth L. Tung

**Affiliations:** 1 Pritzker School of Medicine, University of Chicago, Chicago, IL USA; 2 Section of General Internal Medicine, University of Chicago, Chicago, IL USA; 3 Section of Cardiology, University of Chicago, Chicago, IL USA; 4 Center for Health and the Social Sciences, University of Chicago, Chicago, IL USA

**Keywords:** Housing, Blood pressure, Health disparities, Social determinants of health, Housing policy, Affordable housing

## Abstract

While there is growing awareness of discriminatory housing policies and their adverse impacts on health, little is known about how housing policy may promote health equity. We focused on the Low-Income Housing Tax Credit (LIHTC), the largest affordable housing program in the United States, and examined how living in neighborhoods with LIHTC housing may moderate associations between elevated blood pressure and race/ethnicity in Chicago, IL. A retrospective cohort included 15,339 adult patients at an academic medical center from 2018 to 2019. We used mixed-effects hierarchal logistic regression models to examine elevated blood pressure (diastolic blood pressure ≥ 90 mmHg or systolic blood pressure ≥ 140 mmHg) as a function of race/ethnicity, LIHTC, and race/ethnicity-LIHTC interaction effects, adjusting for patient- and neighborhood-level covariates. A majority were female (63.2%) and non-Hispanic Black (57.6%), with an average age of 50.2 years (SD 19.1). One in ten (9.9%) patients lived in a census tract with LIHTC. Overall, Black patients had 2.52 times the adjusted odds of elevated blood pressure compared to White patients (95% CI = 2.25–2.82). In models examining the moderating effects of LIHTC, the odds ratio of hypertension in Black patients vs. White patients was reduced from 2.62 to 1.52, representing a 67.9% reduction in the difference between Black and White patients. Notably, Black patients, who have been disproportionately impacted by discriminatory housing policies, were the only group found to have moderating effects associated with LIHTC. Housing policies may mitigate neighborhood effects associated with racial disparity, possibly supporting efforts to achieve health equity.

**Supplementary Information:**

The online version contains supplementary material available at 10.1007/s11524-025-00983-y.

## Introduction

Housing instability is increasingly recognized as a core social determinant of health, yet the burden of housing instability remains high [1], particularly among racially minoritized populations. Black Americans make up 12% of the US population, but account for 37% of people that are unhoused [2]. Only 45% of Black households are homeowners, compared to 75% of White households [3]. These inequities result from systematic exclusion: Housing policies that historically reinforced segregation, disinvestment, and mortgage redlining resulted in devalued homes among Black families while reciprocally building wealth among White families. Meanwhile, neighborhood disinvestment led to a loss of economic vitality and employment opportunities necessary for building healthy communities.

Housing instability is associated with adverse health. Adults experiencing homelessness have up to 70% higher rates of cardiovascular events than the general population [4]. In a study of 10,007 adults in Southeastern Pennsylvania, difficulty paying rent was associated with higher odds of hypertension and related behaviors such as cost-related medication underuse [5]. Beyond the individual level, studies have demonstrated linkages between housing characteristics and racial health disparities at the neighborhood level. Kershaw et al. found that racial hypertension disparities were greater in high-segregation areas, likely resulting from racism [6]. In another study, living in a redlined neighborhood was associated with worse cardiovascular health; however, the association weakened as the neighborhood conditions improved, suggesting that neighborhood effects may mitigate disparities [7]. Despite this growing body of literature, few studies have investigated progressive housing policy and its potential health benefits.

One such program, the Low-Income Housing Tax Credit (LIHTC), is the largest source of affordable housing in the United States. Created in 1986, the program incentivizes private-sector creation or rehabilitation of affordable housing. Through a competitive process, states distribute tax credits to housing developers, who must maintain a proportion of low-income units. Applications are evaluated using a Qualified Allocation Plan (QAP), which varies by state. LIHTC developments were traditionally located in high-poverty areas with a high proportion of racially minoritized residents [8]. However, concerns emerged regarding whether LIHTC concentrated poverty, which culminated in the 2015 Supreme Court case *Texas Department of Housing and Community Affairs v. Inclusive Communities Project, Inc*. Since the decision, states have incentivized LIHTC developments in high-income “opportunity” areas or have required proposals in low-income neighborhoods to have concomitant community revitalization plans. These community revitalization plans often include commitments for obtaining investments for infrastructure, transportation, open spaces, or commercial amenities [9]. For example, 17 states require specific investment in non-housing development and infrastructure. Twenty-three states require goals with measurable outcomes. Other requirements may include geographic boundaries or a description of the existing community infrastructure.

Prior studies have examined the health benefits of living in LIHTC housing with mixed results. Gensheimer et al. examined national data and found that low-income individuals living in LIHTC housing versus those who did not were more likely to be insured and have preventive healthcare, but also reported worse health status and frequent emergency department use [8]. In a similar study, low-income children living in LIHTC housing were more likely to have a recent well-child or dental visit, but also reported higher rates of asthma [10]. At the state level, increased LIHTC availability has been associated with decreased intimate partner violence-related homicide [11] and child abuse [12].

However, fewer studies have examined neighborhood-level health benefits of LIHTC. Some theorize that changing neighborhoods through affordable housing development may positively impact health, especially in the context of community revitalization plans. For instance, community revitalization efforts have paired affordable housing development with greenspace and transit development [13], which have been independently shown to improve various health outcomes [14, 15]. One study found that, compared to traditional affordable housing, LIHTC was associated with higher population growth and more commercial and transportation uses [16]. Other studies have documented improvements in walkability and green spaces, with implications for increased physical activity [16]. However, no studies have explicitly examined whether having LIHTC in the neighborhood is associated with benefits for cardiovascular health—a primary contributor to mortality differences between Black and White Americans [17].

The objective of this study was to investigate whether living in a neighborhood with LIHTC was associated with the reduction of racial disparities in elevated blood pressure. Our approach was grounded in Swope and Hernandez’s conceptual model [18], which theorizes four pillars linking housing and health: housing condition and quality, housing affordability, residential stability, and neighborhood factors. We theorized that LIHTC development may improve both residential stability and neighborhood factors, which can, in turn, improve health. For example, high eviction rates have been associated with higher residential turnover, lower social cohesion, and higher cardiovascular disease risk [19]. Alternatively, LIHTC may revitalize a neighborhood by attracting resources to historically disinvested communities. 

Finally, we conceptualized race as an indirect measure of racism [20, 21]. Chronic exposure to racism has been associated with hypertension [21], with scholars positing chronic stress and allostatic load as mechanisms of pathophysiologic dysregulation. As such, a longstanding history of unfair housing policies may be a lever of chronic stress and racism. Taken together, we hypothesized that living in a neighborhood with LIHTC may be associated with a reduction in racial disparities in elevated blood pressure, perhaps by improving residential stability, attracting neighborhood resources to historically disinvested communities, and reducing chronic allostatic load.

## Methods

### Data

We conducted a retrospective cross-sectional study of adult outpatients at an urban academic medical center in Chicago, IL from 2018 to 2019 to examine whether LIHTC housing moderated associations between race/ethnicity and elevated blood pressure. Patients were included if they had a residential address in Chicago and at least one outpatient encounter. For housing data, we utilized a database of LIHTC developments maintained by the U.S. Department of Housing and Urban Development. We included LIHTC developments placed into service from 2009 to 2019 in Chicago, IL. For neighborhood characteristics, we used data from the 2015–2019 American Community Survey (ACS) 5-year estimates. 

We additionally conducted secondary analyses to examine whether LIHTC housing was associated with higher access to health-promoting resources in the neighborhood. For neighborhood resources, we used 2017 data from the National Neighborhood Data Archive (NaNDA) [22], the most recent year published of the publicly available dataset. This study followed the Strengthening the Reporting of Observational Studies in Epidemiology (STROBE) reporting guidelines for observational studies [23]. This study was approved with a waiver of informed consent by the University of Chicago Institutional Review Board.

### Main Measures

The primary dependent variable was elevated blood pressure, obtained from electronic health records. We used objective blood pressure measurements instead of diagnostic codes to limit unmeasured confounding due to underdiagnosis or incomplete electronic health records. Thus, our variable does not necessarily reflect a clinical diagnosis of hypertension but includes all blood pressure measurements for a given individual, with variation in diagnosis, treatment, and control over time. We defined elevated blood pressure as a diastolic blood pressure ≥ 90 mmHg or systolic blood pressure ≥ 140 mmHg, i.e., stage II hypertension as defined by the Joint National Committee (JNC) [24]. We additionally conducted sensitivity analyses defining elevated blood pressure at lower (≥ 130/80) and higher (≥ 160/100) thresholds, based on clinically meaningful criteria.

The primary independent variable was self-reported race/ethnicity. We created a categorical variable for race/ethnicity, which included White (referent), non-Hispanic Black, Hispanic or Latine, unknown/patient declined, or other. The primary moderator of interest was the presence of LIHTC. We created a binary variable indicating whether patients lived in a census tract with or without LIHTC units. We incorporated this variable into the model as an interaction term with race/ethnicity. To account for variability in development size, we also used the median number of LIHTC units as a cutoff and conducted analyses comparing no LIHTC units, < 90 LIHTC units, or ≥ 90 LIHTC units in a census tract.

We adjusted for patient age, sex, insurance status (self-pay, Medicaid/dual eligible, Medicare, private), earliest year of LIHTC placement (2009–2019), and tract-level poverty (defined as > 20% of households below the federal poverty line [FPL]) [25]. Adjustment for tract-level poverty was necessary to address confounding related to any preferential allocation of LIHTC based on neighborhood socioeconomic status, although LIHTC developments were nearly evenly distributed across poor and non-poor neighborhoods during the study period (52.2% versus 47.8%, *p* = 0.11). We initially included neighborhood racial composition as a theoretical confounder, but multicollinearity testing demonstrated a variance inflation factor of 5.61 for Black patient race and Black neighborhood racial composition. Neighborhood racial composition was thus removed from final models.

### Statistical Analysis

Descriptive statistics were calculated for all patients and census tracts. This study used mixed-effects hierarchal logistic regression to examine elevated blood pressure as a function of race/ethnicity (model 1) and race/ethnicity-LIHTC as a multiplicative interaction effect (model 2), nested at tract and patient levels. All models adjusted for the aforementioned confounders.

Analyses were conducted using Stata IC, version 17 (StataCorp LLC). Maps were created using ArcGIS Pro, version 2.2.0.

### Neighborhood-Level Resources

A secondary analysis utilized information from NaNDA, which included tract-level counts of ambulatory health care, grocery stores, gyms/fitness centers, and social services. We examined subtypes within social services, including services for children/youth (e.g., adoption centers, foster care placement services, youth centers), seniors/people with disabilities (e.g., senior centers, adult day care centers, disability support groups), individuals/families (e.g., suicide crisis centers, substance use disorder self-help centers), and vocational services (e.g., job training and counseling). For each resource type, we created quartiles using the number of resources in each tract to describe resource availability as scarce, low, medium, or high within each tract.

We used ordinal logistic regression to analyze neighborhood resources as a function of LIHTC. The dependent variable was a 4-level ordinal variable of the level of resource availability (described above). The independent variable was a binary variable indicating whether LIHTC units were present or absent in a census tract. We adjusted for tract-level age (mean), sex (% male), poverty (binary; > 20% households below the federal poverty level), and racial/ethnic composition. For tract-level racial/ethnic composition, we created a categorical variable which classified tracts as majority Black, Hispanic/Latine, White, or “other” race/ethnicity, with more than 50% of a single race/ethnicity comprising a majority. 

## Results

Data from 15,339 patients were included in the study, representing 161,435 outpatient encounters (Table 1). The average age was 50.2 years (SD 19.1 years). Patients were predominantly female (63.2%) and non-Hispanic Black (57.6%). About half (48.4%) of patients were insured by Medicare and/or Medicaid, and half (47.6%) were privately insured. Approximately half (54.8%) lived in a census tract with > 20% of households below the FPL [25]. One in ten (9.9%) patients in our sample lived in a census tract with LIHTC. Among 161,435 patient encounters, 33.2% had a systolic blood pressure (SBP) ≥ 140 or diastolic blood pressure (DBP) ≥ 90; 11.1% had SBP ≥ 160 or DBP ≥ 100. In our sample, 55.9% of patients had SBP ≥ 130 or DBP ≥ 80, which is higher than the national prevalence of hypertension (49.6%) in 2017–2018 [26].

**Table 1:** (*N* = 15,339) Patient characteristics in the study sample

Patient characteristics	Number	Percent
Age (years)		
Younger than 35	4399	28.7
35–49	3471	22.6
50–64	3289	21.4
65–79	3071	20.0
80 or older	1109	7.2
Female	9691	63.2
Race/ethnicity		
Non-Hispanic White	3618	23.6
Non-Hispanic Black	8839	57.6
Hispanic/Latine	657	4.3
Other	1281	8.4
Unknown/patient declined	944	6.2
Neighborhood characteristics		
> 20% households in poverty	8408	54.8
LIHTC present	1514	9.9
Insurance status		
Self-pay	605	3.9
Medicaid/dual eligible	5401	35.2
Medicare	2025	13.2
Private	7308	47.6
Blood pressure (*N* = 161,435 patient encounters)		
≥ 130/80	90,241	55.9
≥ 140/90	53,552	33.2
≥ 160/100	17,925	11.1

**Fig. 1:**
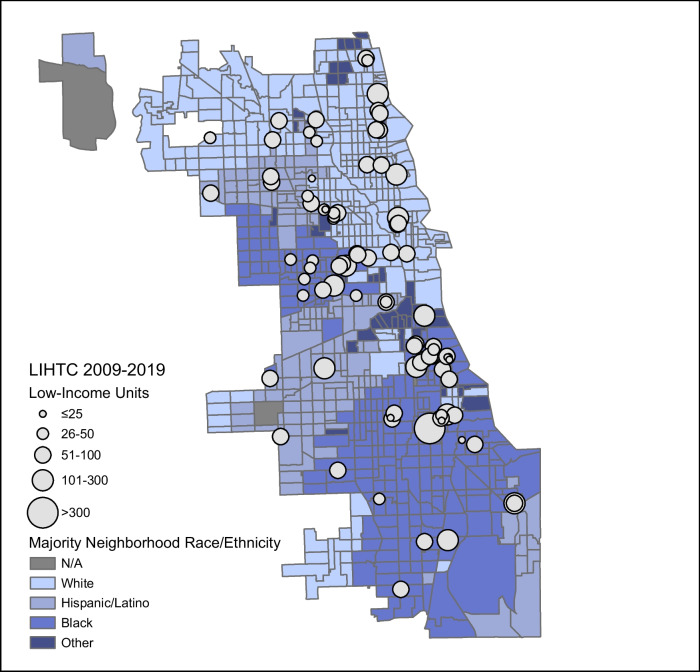
Map of Low-Income Housing Tax Credit (LIHTC) units and majority racial/ethnic makeup of each census tract in Chicago, 2009–2019

We observed clustering of LIHTC housing in Black-majority census tracts (Fig. 1). Although Black-majority census tracts made up 33.7% of census tracts without LIHTC, they accounted for 47.8% of census tracts with LIHTC (*p* = 0.04; data not shown). We additionally observed high levels of residential segregation by race. The percentage of Black patients living in a Black-majority neighborhood was 88.0%. Among those living in neighborhoods with LIHTC, 93.0% of Black residents lived in Black-majority neighborhoods, while that number was 87.3% in neighborhoods without LIHTC (*p* < 0.001; data not shown).

In the study population, 54.8% of patients lived in a neighborhood with > 20% of households below the federal poverty line, compared to 20.1% across Chicago census tracts. Among census tracts in our sample (*N* = 279), we also observed that 18.2% (*n* = 6) of census tracts with LIHTC housing had improvements in neighborhood poverty during the study period (2010–2019), compared to 9.4% (*n* = 23) of census tracts without LIHTC; however, this difference was not statistically significant due to the small sample size (*p* = 0.119) (Table S1).

### Elevated Blood Pressure, LIHTIC, and Race/Ethnicity

In models examining elevated blood pressure (BP ≥ 140/90) as a function of race/ethnicity (Table 2, Model 1), Black patients had 2.52 times the odds of elevated blood pressure compared to White patients (95% CI = 2.25–2.82), adjusted for patient age, sex, insurance status, earliest year of LIHTC placement, and tract-level poverty. Given recent changes in hypertension guidelines, we conducted sensitivity analyses for both lower (BP ≥ 130/80, Table S2) and higher (BP ≥ 160/100, Table S3) thresholds, which also found associations between race/ethnicity and elevated blood pressure.

**Table 2:** (*N* = 15,339) Associations of elevated blood pressure with race/ethnicity and the Low-Income Housing Tax Credit (LIHTC)

	Adjusted odds ratio^a^	
	Model 1: race/ethnicity	Model 2: race/ethnicity-LIHTC interaction
Race/ethnicity		
Non-Hispanic White	Ref	Ref
Non-Hispanic Black	2.52****	2.62****
Hispanic/Latine	1.22*	1.22*
Other race/ethnicity	0.83**	0.86*
Unknown/patient declined	1.31***	1.29***
LIHTC	-^b^	1.07
Race/ethnicity × LIHTC		
Non-Hispanic White	Ref	Ref
Non-Hispanic Black	-^b^	0.58***
Hispanic/Latine	-^b^	0.85
Other race/ethnicity	-^b^	0.58
Unknown/patient declined	-^b^	1.02

^a^Mixed-effects hierarchical regression models were nested at the census tract and patient level and adjusted for patient age, sex, insurance status, neighborhood poverty, and earliest year of LIHTC placement 2009–2019

^b^The model did not include this variable

^*^p < 0.1, **p < 0.05, ***p < 0.01, ****p < 0.001

In models examining moderating effects of LIHTC, we also observed a racial disparity. In census tracts without LIHTC, Black patients had higher odds of elevated blood pressure compared to White patients (aOR = 2.62, 95% CI = 2.34–2.94). However, there was a significant race-LIHTC interaction (Table 2, Model 2), such that Black patients in neighborhoods with LIHTC had a lower odds of elevated blood pressure from 2.62 to 1.52 (Table 3), representing a 67.9% reduction in the difference between Black and White patients (calculation: 1 − [1.52 − 1]/[2.62 − 1]). In sensitivity analyses, we found race-LIHTC interaction effects for Black patients at the lower BP threshold (aOR = 0.54, 95% CI = 0.38–0.78) but not at the higher BP threshold (aOR = 0.72, 95% CI = 0.42–1.21).

**Table 3:** (*N* = 15,339) Associations of elevated blood pressure with race/ethnicity stratified by Low-Income Housing Tax Credit (LIHTC) group

	Adjusted odds ratio^a^	
	Model 2: race/ethnicity-LIHTC interaction	
	No LIHTC	LIHTC^a^
Non-Hispanic White	1.00 (Ref.)	1.00 (Ref.)
Non-Hispanic Black	2.62	1.52
Hispanic/Latine	1.22	1.04
Other race/ethnicity	0.86	0.50
Unknown/patient declined	1.29	1.32

^a^Odds ratios for patients living in tracts without LIHTC are the main effects for race/ethnicity in Table 2. Odds ratios for patients living in tracts with LIHTC were calculated by multiplying these main effects for race/ethnicity by the race/ethnicity-LIHTC interaction terms presented in Table 2. For example, for non-Hispanic Black patients living in tracts with LIHTC, the odds ratio was calculated using 2.62 * 0.58 = 1.52

A reduction in racial disparities was preserved in analyses accounting for variability in LIHTC development size. The interaction between Black race and census tracts with ≥ 90 LIHTC units was 0.49 (95% CI = 0.30–0.81, *p* = 0.05), while that for < 90 LIHTC units was 0.60 (95% CI = 0.37–0.98, *p* = 0.04).

In secondary analyses (*N* = 798 census tracts), we investigated associations between LIHTC and the level of available health-promoting resources in the neighborhood (Table 4). Census tracts with LIHTC had 2.69 higher odds of having a higher level of available services for seniors and people with disabilities (95% CI = 1.43–5.07), and 1.70 times higher odds of having a higher level of available services for individuals and families (95% CI = 1.07–2.69). We observed no significant associations between LIHTC and other resource types.

**Table 4:** (*N* = 798) Associations of the level of neighborhood resource availability and the Low-Income Housing Tax Credit (LIHTC)

	Unadjusted odds ratio	Adjusted odds ratio^a^
Ambulatory health care services	0.67*	0.97
Grocery stores	0.76	0.76
Gyms/fitness centers	0.61*	0.86
Social services	1.39	1.33
Child/youth services	0.83	0.76
Elder/disability services	2.26***	2.69***
Individual/family services	1.60**	1.70**
Vocational relief services	1.57	1.50

^a^Ordinal logistic regression models adjusted for census tract-level age (mean), sex (% male), poverty (binary; > 20% households below the federal poverty level), and racial/ethnic composition. For tract-level racial/ethnic composition, we created a categorical variable which classified tracts as majority Black, Hispanic/Latine, White, or “other” race/ethnicity, with more than 50% of a single race/ethnicity comprising a majority. The dependent variable was defined using quartiles for the number of resources in each census tract by resource type; the independent variable was defined as the presence or absence of LIHTC housing in each census tract.

^*^p < 0.1, **p < 0.05, ***p < 0.01, ****p < 0.001

## Discussion

In this cross-sectional study, we found that living in neighborhoods with LIHTC corresponded with a 67.9% reduction of the difference in odds of elevated blood pressure between Black and White patients. Prior studies have documented racial disparities in hypertension [20], with hypertension being the largest contributor to racial disparities in cardiovascular mortality [17]. Our study extends this work by examining whether affordable housing policy is associated with differences in these racial disparities. Our findings suggest that neighborhoods with LIHTC development may be associated with a smaller racial disparity in elevated blood pressure among Black patients. 

While not causal in nature, we did find robust associations, supporting theories that affordable housing may possibly reinforce pro-health neighborhood effects—even among residents who do not live in affordable housing [18]. Notably, larger developments, which would presumably have a larger imprint on the community, appeared to be associated with larger reductions in the disparity (Table S4). We additionally tested whether living in neighborhoods with LIHTC was associated with increased access to health-promoting resources, which have been shown to improve hypertension, obesity, and other chronic health conditions [27]. We found that LIHTC neighborhoods had a higher availability of some social services, but not ambulatory healthcare, grocery stores, or fitness centers. This finding appears consistent with previous reports that the majority of Chicago LIHTC developments have been in high-poverty neighborhoods. Federal and state programs often emphasize social service development in high-poverty census tracts [28]. Meanwhile, pro-health businesses from the private sector, including for-profit clinics and hospitals, may still be disincentivized to invest in high-poverty neighborhoods with little potential for financial gains [29].

Census tracts that had LIHTC developments also had higher concentrations of Black residents than those without LIHTC developments (93.0% vs. 87.3%). Prior work has found that Black and Latinx LIHTC tenants cannot always “move to neighborhoods with more opportunity” (lower poverty, better air quality, access to jobs/transit and high-performing schools) and often remain in communities similar to non-LIHTC Black and Latinx renters [30]. Although the net impact of locating LIHTC housing in low-income and historically segregated areas is controversial, our study supports the possibility that recent LIHTC allocation priorities may support community revitalization, addressing structural racism instead of race. Forty-two states, including Illinois [31], now require LIHTC proposals in low-income neighborhoods to include community revitalization plans [9], which may counteract structural racism and cultivate health equity without breaking up communities. However, there is significant heterogeneity in revitalization plans between states, and one report in Connecticut noted limited enforcement of such plans after approval [32]. Given this context, LIHTC revitalization plans must be made thoughtfully and with accountability in mind.

Our findings have several policy implications. Notably, the moderating effect was only present for Black patients, who have been disproportionately impacted by adverse housing policies. Such findings suggest that people living in neighborhoods historically most impacted by housing inequity may gain the most from affordable housing development. Future policies may redress discriminatory housing policy by directly aiding affected residents. For example, Evanston, IL, allocated $10 million to be disbursed in cash payments to individual residents affected by racial redlining [33].

Our study also reinforces the potential role of affordable housing policy in promoting community health. Housing instability in neighborhoods, even if not personally experienced, can be associated with toxic stress. For example, residents in neighborhoods with high eviction rates have greater exposure to crime [34], which has been associated with increased blood pressure and cardiovascular-related hospital admission [35]. High housing turnover and vacancy can also lead to neighborhood blight, which can adversely impact cardiovascular health. In one Philadelphia-based study, remediating neighborhood blight was associated with reductions in cardiovascular reactivity [36]. With thoughtful development, LIHTC housing can stabilize residential turnover, improve neighborhood conditions, and promote community resources [37]. Expansion of community revitalization requirements could include robust strategies to promote health in neighborhoods and ensure adequate access to health-promoting resources.

This study has several limitations. Given that the effects of the built environment on health outcomes likely take years to decades to manifest, the brief study period (2018–19) may not capture the full effect of LIHTC on blood pressure. Additionally, because our analysis used cross-sectional data, we cannot fully describe the causal relationship between LIHTC and blood pressure. Even if not causal, our findings suggest that LIHTC and reduced blood pressure disparities are at least co-located, raising important questions about the role of housing policy in neighborhood development and revitalization. Further investigation is needed to more fully examine whether residential stability, increased resource allocation, and/or pro-social processes are contributing to associations between housing policy and health.

We analyzed housing at the census tract rather than the individual level. However, one of our goals was to examine LIHTC housing as a neighborhood effect. We acknowledge that using blood pressure data from electronic health records is nonrandom, as people who seek healthcare may do so due to underlying illness and other factors. We did not account for antihypertensive medications due to incomplete data and difficulty capturing real-world use. Thus, our measurement of elevated blood pressure could include both untreated and inadequately treated hypertension. 

We were limited in power to examine neighborhood poverty as a mediator of LIHTC and blood pressure disparities. Although we observed that six majority-Black census tracts with LIHTC improved their poverty status between 2010 and 2019, these improvements in neighborhood poverty were not statistically significant due to small sample size (Table S1). This was a single-center study in Chicago and may not be generalizable to other geographic areas. Qualified Allocation Plans for LIHTC developments are state-specific, so allocation may vary outside Illinois. Moreover, our study does not consider other affordable housing programs. Finally, although we adjusted for several covariates, residual confounding may be present. However, we are reciprocally sensitive to over-adjustment bias in studies of structural risk and were careful to exclude variables that may be intermediate outcomes rather than confounders. 

## Conclusion

Among outpatients at a large academic health center in Chicago, we found that living in neighborhoods with LIHTC housing corresponded with a 67.9% reduction of the Black-White difference in odds of elevated blood pressure. This study suggests that LIHTC development, and its potential for neighborhood or community revitalization, may be associated with reduced racial health disparities. Our findings support the relevance of incorporating health and community revitalization priorities into affordable housing policies. Aligning healthcare goals that address individual social risk factors (e.g., housing instability) with broader policies that mitigate the historical effects of racism (e.g., racial redlining) may be needed to improve health equity for racially minoritized communities.

## Supplementary Information

Below is the link to the electronic supplementary material.

Supplementary file1 (DOCX 21 KB)

## Data Availability

Individual-level clinical data are identified and therefore cannot be shared. However, all source code can be shared upon request.
