# Supplementary material for: Moderating Effects of the Low-Income Housing Tax Credit on Associations Between Race and Elevated Blood Pressure in Chicago
Source: J Urban Health. 2025 Jun 18;102(3):520–9. doi: 10.1007/s11524-025-00983-y (PMC12279677; doi:10.1007/s11524-025-00983-y)
Supplement: Supplementary file 1 — Supplementary file1 (DOCX 21 KB) [file 11524_2025_983_MOESM1_ESM.docx]

Table S1. **Majority-Black census tracts with change in neighborhood poverty status 2010-2019 by Low-Income Housing Tax Credit (LIHTC) allocation**. *N* = 279 majority-Black census tracts; *p* = 0.119.

|  | **Tracts without LIHTC units** | **Tracts with LIHTC units** | | **Total** |
| --- | --- | --- | --- | --- |
| Tract without reductions in neighborhood poverty 2010-2019 | 223 (90.7%) | | 27 (81.8%) | 250 (89.6%) |
| Tract with improvements in neighborhood poverty | 23 (9.4%) | | 6 (18.2%) | 29 (10.4%) |
| Total | 246 (100%) | | 33 (100%) | 279 (100%) |

Table S2. **Sensitivity analysis of blood pressure ≥130/80: Associations of race/ethnicity and the Low-Income Housing Tax Credit (LIHTC) with elevated blood pressure in Chicago, Illinois 2018–2019.** *N* = 15,339.

|  | **Adjusted odds ratio^a^** | |
| --- | --- | --- |
|  | **Model 1: race/ethnicity** | **Model 2: race/ethnicity-LIHTC interaction** |
| Race/ethnicity |  |  |
| Non-Hispanic White | Ref | Ref |
| Non-Hispanic Black | 2.11**** | 2.21**** |
| Hispanic/Latinx | 1.05 | 1.08 |
| Other race/ethnicity | 0.83** | 0.84** |
| Unknown/Patient declined | 1.04 | 1.03 |
| LIHTC | -^b^ | 1.28 |
| Race/ethnicity × LIHTC |  |  |
| Non-Hispanic White | Ref | Ref |
| non-Hispanic Black | -^b^ | 0.54*** |
| Hispanic/Latinx | -^b^ | 0.70 |
| Other race/ethnicity | -^b^ | 0.74 |
| Unknown/Patient declined | -^b^ | 0.94 |

^a^Mixed-effects hierarchical regression models implemented nested at the census tract and patient level and adjusted for patient age, sex, insurance status, neighborhood poverty, and earliest year of LIHTC placement 2009-2019.

^b^The model did not include this variable.

**p* < 0.1 ***p* < 0.05 ****p* < 0.01 *****p* < 0.001

Table S3. **Sensitivity analysis of blood pressure ≥160/100: Associations of race/ethnicity and the Low-Income Housing Tax Credit (LIHTC) with elevated blood pressure in Chicago, Illinois 2018–2019.** *N* = 15,339.

|  | **Adjusted odds ratio^a^** | |
| --- | --- | --- |
|  | **Model 1: race/ethnicity** | **Model 2: race/ethnicity-LIHTC interaction** |
| Race/ethnicity |  |  |
| Non-Hispanic White | Ref | Ref |
| Non-Hispanic Black | 2.64**** | 2.70**** |
| Hispanic/Latinx | 1.31** | 1.29* |
| Other race/ethnicity | 0.96 | 0.99 |
| Unknown/Patient declined | 1.55**** | 1.47*** |
| LIHTC | -^b^ | 0.96 |
| Race/ethnicity × LIHTC |  |  |
| Non-Hispanic White | Ref | Ref |
| non-Hispanic Black | -^b^ | 0.72 |
| Hispanic/Latinx | -^b^ | 1.11 |
| Other race/ethnicity | -^b^ | 0.60 |
| Unknown/Patient declined | -^b^ | 1.46 |

^a^Mixed-effects hierarchical regression models implemented nested at the census tract and patient level and adjusted for patient age, sex, insurance status, neighborhood poverty, and earliest year of LIHTC placement 2009-2019.

^b^The model did not include this variable.

**p* < 0.1 ***p* < 0.05 ****p* < 0.01 *****p* < 0.001

Table S4. **Sensitivity analysis by development size: Associations of race/ethnicity and the Low-Income Housing Tax Credit (LIHTC) with elevated blood pressure in Chicago, Illinois 2018–2019.** *N* = 15,339.

|  | **Adjusted odds ratio^a^** | |
| --- | --- | --- |
|  | **Model 1: race/ethnicity** | **Model 2: race/ethnicity-LIHTC interaction** |
| Race/ethnicity |  |  |
| Non-Hispanic White | Ref | Ref |
| Non-Hispanic Black | 2.52**** | 2.21**** |
| Hispanic/Latinx | 1.22* | 1.08 |
| Other race/ethnicity | 0.83** | 0.84** |
| Unknown/Patient declined | 1.31*** | 1.03 |
| LIHTC |  |  |
| <90 units | -^b^ | 1.25 |
| ≥90 units | -^b^ | 1.42 |
| Race/ethnicity × LIHTC (<90 units) |  |  |
| Non-Hispanic White | Ref | Ref |
| non-Hispanic Black | -^b^ | 0.60** |
| Hispanic/Latinx | -^b^ | 0.55 |
| Other race/ethnicity | -^b^ | 1.17 |
| Unknown/Patient declined | -^b^ | 1.41 |
| Race/ethnicity × LIHTC (≥90 unites) |  |  |
| Non-Hispanic White | Ref | Ref |
| non-Hispanic Black | -^b^ | 0.49*** |
| Hispanic/Latinx | -^b^ | 0.81 |
| Other race/ethnicity | -^b^ | 0.47* |
| Unknown/Patient declined | -^b^ | 0.61 |

^a^Mixed-effects hierarchical regression models nested observations at the census tract and patient level and adjusted for patient age, sex, insurance status, neighborhood poverty, and earliest year of LIHTC placement 2009-2019.

^b^The model did not include this variable.

**p* < 0.1 ***p* < 0.05 ****p* < 0.01 *****p* < 0.001.
